# SIADH as an uncommon presentation of olfactory neuroblastoma: a case-based overview

**DOI:** 10.1530/EO-25-0021

**Published:** 2025-07-07

**Authors:** Solene Papart, Adrian F Daly, Elettra Bianchi, Alexandre Jadoul, Gilles Reuter, Olivier Bouchain, Patrick Pétrossians, Albert Beckers

**Affiliations:** ^1^ Department of Opthalmology, Centre Hospitalier Universitaire de Liège, University of Liège, Liège, Belgium; ^2^ Department of Endocrinology, Centre Hospitalier Universitaire de Liège, University of Liège, Liège, Belgium; ^3^ Department of Pathology, Centre Hospitalier Universitaire de Liège, University of Liège, Liège, Belgium; ^4^ Department of Nuclear Medicine, Centre Hospitalier Universitaire de Liège, University of Liège, Liège, Belgium; ^5^ Department of Neurosurgery, Centre Hospitalier Universitaire de Liège, University of Liège, Liège, Belgium; ^6^ Department of Otorhinolaryngology, Centre Hospitalier Universitaire de Liège, University of Liège, Liège, Belgium

**Keywords:** olfactory neuroblastoma, esthesioneuroblastoma, SIADH, hyponatraemia, paraneoplastic

## Abstract

**Background:**

Olfactory neuroblastoma (ONB) is a rare malignant neuroectodermal tumour that usually arises in the nasal cavity, typically presenting with unilateral nasal obstruction and epistaxis. In rare instances, ONB can manifest as a paraneoplastic syndrome of inappropriate antidiuretic hormone (SIADH) secretion, leading to hyponatraemia.

**Case presentation:**

We describe a 42-year-old woman with a 4-year history of cyclical, symptomatic hyponatraemia characterised by intermittent episodes of dizziness, severe headaches and marked fatigue, initially without overt nasal or otolaryngological symptoms. Investigations confirmed SIADH, yet repeated imaging (including thoracic CT and FDG PET-CT) failed to identify a source. Eventually, a ^68^Ga-DOTANOC PET-CT revealed an isolated lesion in the left ethmoid region. Surgical resection via an endoscopic approach confirmed the diagnosis of a Hyams grade 1 ONB. Following complete tumour removal, the patient’s hyponatraemia resolved.

**Literature review:**

ONB accounts for <3% of nasal tumours and can present with non-specific symptoms, delaying diagnosis. Most patients have a typical combination of nasal obstruction and epistaxis, but paraneoplastic SIADH is reported in about 2% of cases. Complete surgical excision is the cornerstone of therapy, often accompanied by radiotherapy or chemotherapy for higher-grade lesions. Recent advances suggest that somatostatin receptor imaging and targeted radionuclide therapy might benefit select patients, particularly those with unresectable or recurrent disease.

**Conclusion:**

This case highlights the importance of considering ONB in patients with unexplained SIADH, especially when initial investigations are inconclusive. It also underscores the utility of ^68^Ga-DOTANOC PET-CT in detecting occult neuroendocrine tumours, and reinforces the value of prompt surgical intervention for definitive treatment.

## Introduction

Olfactory neuroblastoma (ONB), also known as esthesioneuroblastoma, is a rare malignant neuroectodermal tumour that arises from the nasal cavity, usually developing in the olfactory placode. ONBs are relatively uncommon, accounting for <3% of nasal tumours (Martel *et al.* 2000). However, this prevalence may be underestimated due to delayed diagnosis (Senchak *et al.* 2012). ONBs have traditionally been considered as having two peaks of frequency, one between 11 and 20 years of age and the other between 50 and 60, although a monophasic distribution peaking in the fifth and sixth decades has also been proposed (Bernard *et al.* 2000, Yin *et al.* 2018). Some series suggest a slight male preponderance (Brisson *et al.* 2021). Tumour behaviour varies from indolent growth with survival of >20 years to highly aggressive ONBs with extensive metastasis and survival limited to a few months (Dupuy *et al.* 2012). Due to its low incidence, the development of management guidelines has been hindered (Theilgaard *et al.* 2003). Nevertheless, most groups agree on the importance of achieving a complete surgical resection (Savelli *et al.* 2016). Surgery is usually accompanied by adjuvant treatment, such as radiotherapy, chemotherapy or, more recently, radioligand therapy (Harvey *et al.* 2017, Brisson *et al.* 2021, Abdelmeguid *et al.* 2022, McMillan *et al.* 2022).

Typical presenting signs of ONB are unilateral nasal obstruction and epistaxis, both of which occur in more than 75% of cases (Bernard *et al.* 2000). However, in most cases, this lesion evolves slowly and is asymptomatic for long periods. Consequently, the above-mentioned nasal signs tend to appear late in the disease. Exceptionally, ONB can be discovered due to the secondary effects of abnormal hormone production by the tumour. Here we describe a patient who had an extended period of recurrent severe hyponatraemia and was diagnosed with the syndrome of inappropriate antidiuretic hormone secretion (SIADH) secondary to an ONB.

## Clinical case

A 42-year-old woman was assessed for cyclical hyponatraemia over the past 4 years. She suffered from acute episodes of hyponatraemia that manifested as dizziness, severe headaches, a feeling of intoxication and severe neck pain. She had no clear otorhinolaryngological symptoms. Some episodes were accompanied by diarrhoea. In her medical history, she had recurrent renal lithiasis, an appendicectomy and migraines. The patient gave written permission for the anonymous details of her case to be published.

**Figure 1:**
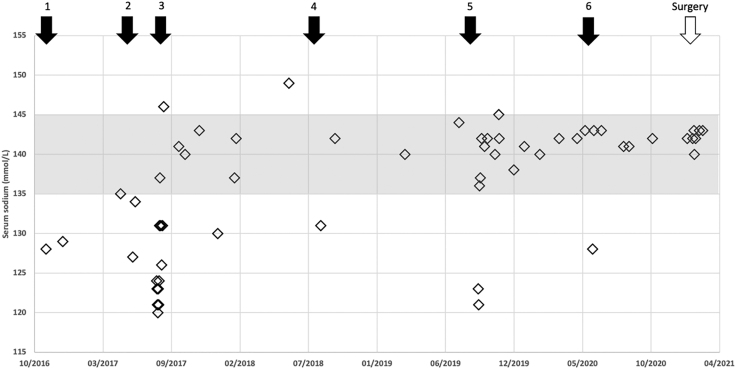
Sodium levels over time, with episodes of severe symptomatic hyponatraemia numbered. Normal range of serum sodium is shown in grey.

As shown in Fig. 1, she had her first episode of hyponatraemia in late 2016. Hyponatraemia was noted during a Streptococcus A infection in October 2016. During that period, the patient underwent a cholecystectomy for acute cholelithiasis. As the hyponatraemia persisted, initial treatment consisted of water restriction (1,500 mL/24 h), NaCl supplementation by gel and urea *per os*. The sodium was stabilised, and the treatment was stopped in March 2017. The patient presented to the emergency department due to a second acute episode of hyponatraemia in May 2017, which led to a resumption of her previous treatment. She relapsed again thereafter, suffering episodes of dizziness, asthenia, headaches, diffuse muscular pain and swelling of the hands and face. Several methods were used to increase plasma sodium, including water restriction (1,000 mL/24 h), NaCl by gel, urea and oral furosemide 40 mg/day. The patient tapered this therapy at the end of 2017 to 20 mg furosemide daily, and fluid restriction was relaxed to 1,500 mL/24 h. The patient had a minor symptomatic hyponatraemic episode in August 2018, after which she resumed furosemide 40 mg daily and tightened fluid restriction to 1,200 mL/24 h. A fourth relapse was documented in September 2019, despite maintaining her furosemide and fluid restriction. Furosemide was replaced with bumetanide 1 mg/day, and fluid restriction was maintained. The patient had a further symptomatic episode in June 2020, with stabilisation of the plasma sodium being achieved by an increase of bumetanide to 1.5 mg/day and a decrease of the water intake to 1,000 mL/24 h for a few days. Despite initial improvement, she reported subsequent cyclical symptoms with an interval of about 10 days, which were characterised by muscle pain, marked asthenia, headache that was worse at night, a feeling of intoxication and a swollen face and hands for 3–4 days, always ending with an episode of liquid stools. In addition, the patient reported weight gain of more than 14 kg, despite a balanced diet.

At that time, she was referred to the Endocrinology Department of the University Hospital of Liège. She was admitted for re-evaluation. During her first hyponatraemic episode in 2016, SIADH was considered, but the aetiology remained unknown, despite an extensive evaluation including thoracic CT scan, pituitary MRI and fluorodeoxyglucose positron emission tomography computed tomography (FDG PET-CT). On the new assessment, the presence of euvolaemic hyponatraemia, plasma and urinary hyperosmolality, in the absence of hypothyroidism or glucocorticoid insufficiency, permitted us to confirm SIADH. Given the unexplained nature of the patient’s chronic, severe SIADH, the possibility of an occult tumoural source was investigated.

**Figure 2:**
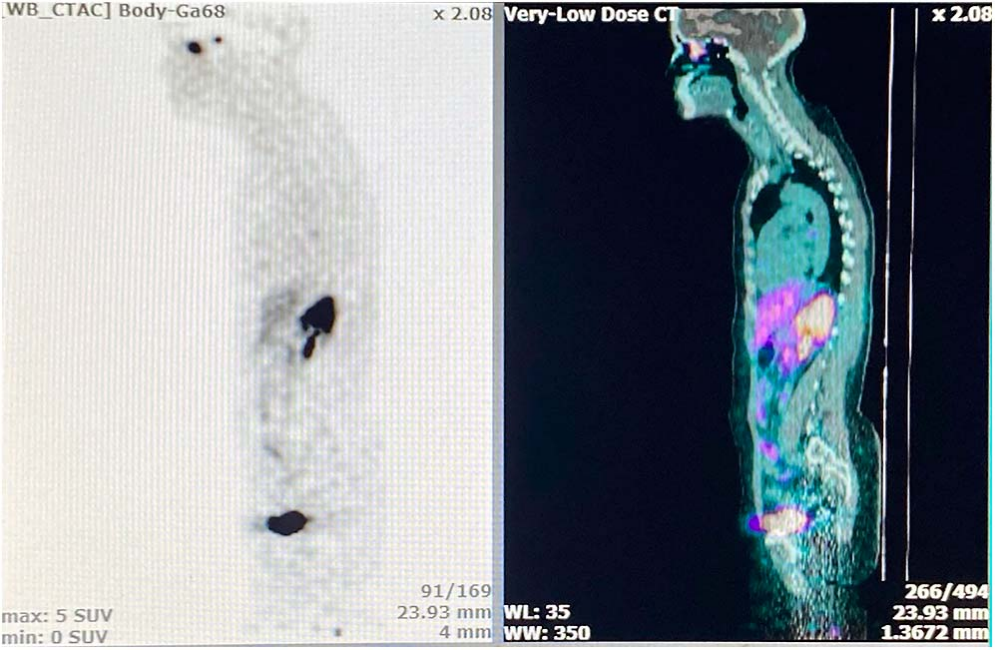
^68^Ga-DOTANOC PET-CT images showing intense hyperfixation projecting to the left ethmoidal cells.

**Figure 3:**
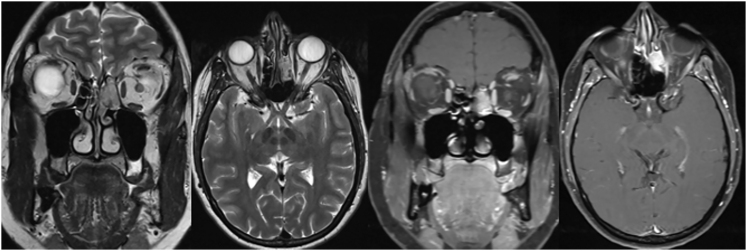
Preoperative MRI shows an intensely arterialised expansive tumour without locally invasive characteristics (21 × 20 × 10.5 mm). It is situated at the left anterior ethmoidal labyrinth, reaching the Onodi cells, then extending under the ethmoidal fovea and inside the left middle concha in contact with the nasal septum. There is no modification of the anterior floor of the skull base.

A series of radiological imaging studies were performed and historical images from other centres were collected and reviewed. Gallium-68 DOTA-1-Nal3-octreotide positron emission tomography computed tomography (^68^Ga-DOTANOC PET-CT) was performed to investigate for a neuroendocrine tumour and revealed an isolated lesion in the left ethmoidal region (Fig. 2). An MRI allowed a more precise characterisation of this lesion, which showed an expansive, intensely arterialised tumour without local invasion. The lesion, measuring 21 × 20 × 10.5 mm, was in the left anterior ethmoidal labyrinth, reaching the Onodi air cells, extending under the ethmoidal fovea and medial to the left middle turbinate. The lesion was in contact with the nasal septum without alteration of the anterior floor of the skull base (Fig. 3).

Given the tumour characteristics and the intermittent severe SIADH, a working diagnosis of ONB was made, and she was referred for treatment to neurosurgery and otorhinolaryngology. At operation, the lesion was removed as a gross-total *en-bloc* resection via an endoscopic approach after partial embolisation. Vascularisation of the ethmoid depends on the ophthalmic artery and the sphenopalatine artery; hence, embolisation could only be performed on the sphenopalatine artery in consideration of the visual risks involved in embolising the ophthalmic artery. The tumour was adherent to the cribriform plate and emerged from olfactory fibres. The olfactory fibres were identified and cut close to the olfactory bulb. The skull base defect was reconstructed with middle turbinate mucosa. The postoperative course was uneventful, and the patient did not experience a cerebrospinal fluid leak. After the resection of the ONB, the patient’s SIADH resolved completely, and she remains asymptomatic 5 years postoperatively, thereby confirming the link between the tumour and the symptomatology (Senchak *et al.* 2012).

**Figure 4:**
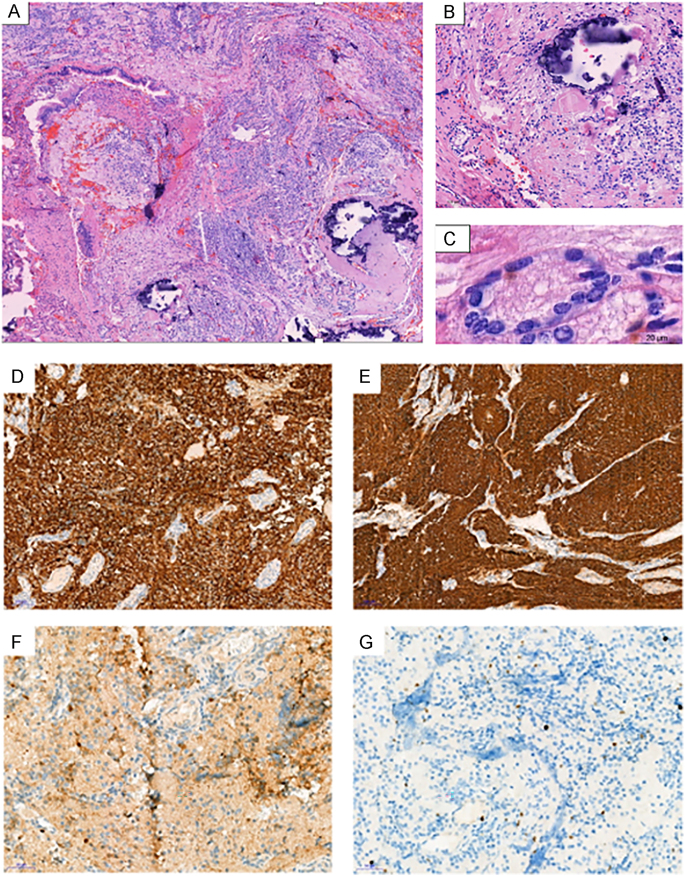
Haematoxylin and eosin staining of resected ONB at (A) low power; (B) high power showing calcifications; (C) a Flexner-Wintersteiner rosette; (D) positivity for synaptophysin; (E) positivity for CD56; (F) negative staining for Cytokeratin AE1/AE3; (G) Ki-67 of 1%.

The pathological study of the surgical specimen (Fig. 4A) confirmed an ONB that was grade 1 according to Hyams’ histological classification. Morphologically, the sinonasal mucosa could be identified, which had haemorrhagic areas and was covered by a well-differentiated respiratory epithelium. The underlying chorion was infiltrated by tumour that was partially calcified (Fig. 4B) and had a solid/lobular architecture consisting of small ‘blue’ cells with scarce cytoplasm and an increased nuclear-cytoplasmic ratio. These cells had round nuclei that were regular in outline, with a ‘salt and pepper’ chromatin appearance typical of cells found in neuroendocrine tumours. In places, the cells formed small Flexner-Wintersteiner rosettes (Fig. 4C). Pseudorosettes or Homer Wright rosettes were not found in this case.

From an immunohistological point of view (Fig. 4), the tumour cells expressed the cytoplasmic markers CD56 and synaptophysin intensely and diffusely. These were slightly positive for calretinin and negative for cytokeratin AE1/AE3 and CD45, which allowed us to confirm the neuroendocrine nature of the tumour. Furthermore, Ki-67 was assessed at 1% in the tumour lesion, which correlates with the slow clinical evolution of this lesion.

## Literature overview

ONB was first described in 1924 by Berger (Berger 1924). Since then, more than 1,200 cases have been published. ONB remains a rare tumour of the nasal cavity that manifests in the majority of cases with non-specific symptoms such as unilateral nasal obstruction, hypoosmia, epistaxis and rhinorrhoea (Bernard *et al.* 2000, Fiani *et al.* 2019). Facial and neck pain are also often reported. Ocular signs, on the other hand, are reported in only 10% of cases; these include exophthalmia, orbital pain, diplopia, loss of visual acuity and ophthalmoplegia (Bouziri *et al.* 2008). While derived from neuroendocrine tissues, only a minority of cases (8%) have associated paraneoplastic hormonal secretion, and only 2% have some form of hyponatraemia-SIADH (Gabbay *et al.* 2013, Fiani *et al.* 2019). There are no known risk factors for ONB, and the presence of SIADH does not seem to be related to tumour severity (Senchak *et al.* 2012, Sheehan & Payne 2016).

In the current case, we describe a biochemically severe presentation of ONB, where the patient suffered from few local tumoural symptoms, and whose clinical features consisted of severe episodic symptomatic hyponatraemia alternating with asymptomatic periods. The slow growth of the tumour in the current case meant that there was no spread to the skull base, orbits or cavernous sinus, which can complicate a successful oncological resection (Bak & Wein 2012). Apart from the severe episodic SIADH as an unusual presenting syndrome, the patient, who was 38 years old at first manifestations of hyponatraemia, fell outside the typical peak age range, which has been reported to be unimodal (5th–6th decades) or bimodal (11–20 years and 50–60 years) (Bernard *et al.* 2000, Yin *et al.* 2018).

SIADH is characterised by hyponatraemia, plasma hypo-osmolarity, euvolaemia and urine hyperosmolarity. The causes of SIADH are legion: in the oncological setting, the most frequently reported paraneoplastic syndrome is related to small cell lung cancer. In our case, the possibility of a lung tumour was excluded by thoracic imaging. The association between an ONB and paraneoplastic SIADH is unusual, although symptomatic disease due to vasopressin excess can precede the diagnosis of ONB by some months (Gray *et al.* 2012, Gabbay *et al.* 2013, Nakano *et al.* 2017, Parrilla *et al.* 2017, Saffarzadeh *et al.* 2023, Daloiso *et al.* 2025). Given the episodic nature of the severe symptoms in the current case, tumoural vasopressin secretion was probably not constant but fluctuated, indicating unstable pathological secretory patterns.

The diagnosis of vasopressin-secreting ONB is mainly based on clinical observation, fixation on ^68^Ga-DOTANOC PET-CT labelling somatostatin subtype 2 receptors (Oskouian *et al.* 2002) and pathological analysis of the resected mass. In our case, the mass was visualised on ^68^Ga-DOTANOC PET-CT but was not visible on FDG. Apart from the slow evolution of the tumour, immunohistochemistry allowed us to differentiate carcinoma from ONB using different markers (Wooff *et al.* 2011). These included ONB positivity for chromogranin, synaptophysin, neuron-specific enolase (NSE) and S100 (in sustentacular cells), and negativity for keratin and Ebstein-Barr virus RNA. Histopathological examination supported the diagnosis of ONB, which was without necrosis or mitosis and was classified as a Hyams grade 1. It presented features of a typical neuroblastoma, being comprised of small round cells, with a ‘salt and pepper’ chromatin-rich nucleus and a characteristic fibrillated background. In places, the cells formed small Flexner-Wintersteiner rosettes, which are seen in only 5% of cases. The more common pseudorosettes or Homer Wright rosettes, which are seen in up to 50% of cases, were not identified (Bak & Wein 2012). Calretinin positivity was a marker favouring ONB over a blue round cell tumour (Wooff *et al.* 2011). In addition, CD45, a marker for lymphoma, was negative.

The prognosis of ONB remains relatively poor despite treatment; overall survival at 5 years is about 70–90%, but recurrence decreases 10-year overall survival to <65% (Abdelmeguid *et al.* 2022, McMillan *et al.* 2022, Choby *et al.* 2023, Ghanem *et al.* 2025). A recent meta-analysis showed, furthermore, that 12% of patients treated for ONB develop distant metastases (Marinelli *et al.* 2018). In the current case, tumoural expansion was relatively slow, and it had not invaded the dura mater or the orbital plate and did not show other forms of locoregional invasion such as cervical lymph involvement. According to Goshtasbi *et al.* (2019), who divided the Hyams ONB classification in a binary fashion into low-grade (Hyams 1 and 2) and high-grade (Hyams 3 and 4), high-grade lesions were twice as likely to develop metastases as low-grade tumours. Survival in the high- and low-grade groups was 54 and 80%, respectively, at 5 years and 36% and 67%, respectively, at 10 years (Goshtasbi *et al.* 2019). These observations are consistent with the results of the largest cohorts of ONB in the literature (Tajudeen *et al.* 2014, Choby *et al.* 2023). Prognosis in the current case should be favourable, therefore, based on the histological (Hyams grade 1) and anatomical (Kadish/modified Kadish stage A) grades (Choby *et al.* 2023). With an estimated Ki-67 of 1%, complete resection and absent lymph node metastases (Oskouian *et al.* 2002, Capelle & Krawitz 2008), there is a good prognosis with a low risk of later recurrence in this case.

The rarity of ONB, with an annual incidence of 0.4 per million (Theilgaard *et al.* 2003), means that few data are available to form a definitive therapeutic consensus. Surgical treatment is considered as the central pillar of treatment of ONB (Savelli *et al.* 2016), with endoscopic surgery being increasingly used in clinical practice, as it has been shown to reduce patient morbidity (Fu *et al.* 2016). However, almost half of the patients have disease recurrence after a 10-year follow-up, implying that a considerable number of patients need additional treatment (Czapiewski *et al.* 2018). Morita *et al.* recommend that patients with high-grade lesions should be treated with surgery and postoperative radiotherapy, and chemotherapy should be considered (Morita *et al.* 1993). ONBs are both chemo- and radiosensitive, but no consensus has been reached on the sequence of administration of surgery and adjuvant therapy (Fiani *et al.* 2019). A systematic review of 700 ONB patients did not identify any survival benefit of adjuvant radiotherapy for affected patients (Kane *et al.* 2010). Similarly, Platek and colleagues (Platek *et al.* 2011), in a review of 511 patients with ONB in the SEER database (1973–2006), concluded that there was no significant improvement in survival at 10 years despite the addition of radiotherapy to surgery. However, other studies show a significant benefit of radiotherapy in terms of distant metastasis-free survival (Ward *et al.* 2009, Marinelli *et al.* 2018). Chemotherapy, on the other hand, shows promising results in patients with high-grade tumours (Hyams grade III and IV) (Porter *et al.* 2008, Sheehan & Payne 2016, Fiani *et al.* 2019). Chemotherapy should therefore be considered on a case-by-case basis as neoadjuvant therapy, especially for advanced grades, but no definitive treatment recommendations have been established (Sheehan & Payne 2016).

Recent advances on the genetic basis of ONB have identified a similarity between ONB and small cell lung cancer, as they share a number of lineage drivers that appear to give rise to various tumoural subtypes (Finlay *et al.* 2024). Like small cell lung cancer and other neuroendocrine-related tumours, peptide receptor radionuclide therapy has been suggested in the context of ONB management (Makis *et al.* 2015, Sabongi *et al.* 2016, Savelli *et al.* 2016, Hasan *et al.* 2020, Ghanem *et al.* 2025). As in our case, other recent studies report somatostatin receptor expression in ONB; this supports the potential utility of somatostatin receptor subtype 2 targeting compounds such as ^177^Lu-octreotate (Lutathera™) in inoperable, incompletely resected or recurrent ONB cases (Czapiewski *et al.* 2018, Gabriel *et al.* 2019).

## Conclusion

Our case underlines that ONB should be considered in patients presenting with SIADH of unknown origin. Its pauci-symptomatic nature and evolution mean that in-depth otorhinolaryngological exploration and subsequent imaging may be necessary to identify an ONB. Moreover, in light of our patient’s history, it is important to highlight the utility of ^68^Ga-DOTANOC PET-CT in exploring this rare nasal neuroendocrine tumour.

## Declaration of interest

The authors declare that there is no conflict of interest that could be perceived as prejudicing the impartiality of the work reported.

## Funding

This work did not receive any specific grant from any funding agency in the public, commercial or not-for-profit sector.

## Author contribution statement

AB conceived the study. SP, EB, AJ, GR, OB and AB collected clinical, biochemical, pathological, radiological and surgical data. SP, AFD, EB, AJ, PP and AB analysed data, conducted the literature review, developed figures and wrote the manuscript. All authors reviewed and approved the final version.
